# Adaptive Two-Step Bearing-Only Underwater Uncooperative Target Tracking with Uncertain Underwater Disturbances

**DOI:** 10.3390/e23070907

**Published:** 2021-07-16

**Authors:** Xianghao Hou, Jianbo Zhou, Yixin Yang, Long Yang, Gang Qiao

**Affiliations:** 1 School of Marine Science and Technology, Northwestern Polytechnical University, Xi’an 710072, China; houxianghao1990@163.com (X.H.); yxyang@nwpu.edu.cn (Y.Y.); lyang@nwpu.edu.cn (L.Y.); 2 College of Underwater Acoustic Engineering, Harbin Engineering University, Harbin 150001, China; qiaogang@hrbeu.edu.cn; 3 Shaanxi Key Laboratory of Underwater Information Technology, Xi’an 710072, China

**Keywords:** bearing-only tracking, two-step filter, adaptive tracking, Kalman filter

## Abstract

The bearing-only tracking of an underwater uncooperative target can protect maritime territories and allows for the utilization of sea resources. Considering the influences of an unknown underwater environment, this work aimed to estimate 2-D locations and velocities of an underwater target with uncertain underwater disturbances. In this paper, an adaptive two-step bearing-only underwater uncooperative target tracking filter (ATSF) for uncertain underwater disturbances is proposed. Considering the nonlinearities of the target’s kinematics and the bearing-only measurements, in addition to the uncertain noise caused by an unknown underwater environment, the proposed ATSF consists of two major components, namely, an online noise estimator and a robust extended two-step filter. First, using a modified Sage-Husa online noise estimator, the uncertain process and measurement noise are estimated at each tracking step. Then, by adopting an extended state and by using a robust negative matrix-correcting method in conjunction with a regularized Newton-Gauss iteration scheme, the current state of the underwater uncooperative target is estimated. Finally, the proposed ATSF was tested via simulations of a 2-D underwater uncooperative target tracking scenario. The Monte Carlo simulation results demonstrated the reliability and accuracy of the proposed ATSF in bearing-only underwater uncooperative tracking missions.

## 1. Introduction

Accurate and robust underwater target tracking and advanced parameter estimation are becoming increasingly important research areas in marine science. Underwater surveillance is a core component of national defense and economic development [1,2]. Good estimates of the motion parameters of an underwater uncooperative target not only can be used to obtain useful prior information about a target but also can provide support for further military or economic actions. Therefore, developing more advanced underwater uncooperative target tracking techniques to more robustly and accurately track the target is of great value.

Two kinds of tracking mechanisms can be applied to underwater target tracking scenarios, namely, active tracking [3,4] and passive tracking [5,6,7]. By utilizing the active sonar systems mounted on facility platforms (usually ship-borne or shore-based platforms), the active tracking system can track an underwater target with high precision in terms of both angle information and range information. However, because the active tracking system depends on the active sonar system, which needs to emit acoustic signals, the tracking system has a high power consumption and is hard to conceal. In addition, due to its implementation on fixed shore-based facility platforms and exposed ship platforms, the active tracking system lacks mobility and the capacity for concealment. However, flexibility and concealment are key considerations for applications in national defense. Therefore, passive tracking systems, which accomplish tracking only via the angle information obtained from the passive radiated noise created by the underwater uncooperative target, have been examined by several researchers [8,9,10]. In the specific underwater tracking scenario in which an underwater target operates at a depth that is far less than the distance between the target and the passive tracking system, passive underwater tracking can be modeled as a 2-D bearing-only tracking (BOT) problem, which is subject to a large number of technical challenges.

According to References [11,12], the underwater 2-D BOT problem is characterized by the inherent challenges in low observability and high nonlinearity. As a result, the tracking system is unobservable when only one fixed observer works during the passive tracking procedure. To ensure that the tracking process is observable and thus to satisfy the requirements of robust estimation, the single observer must maneuver with significantly more agility than that of the target; if this cannot be achieved, more observers are needed [13,14]. Usually, deploying more observers not only saves energy but also allows for concealment. Therefore, the deployment of more than one observer during the target tracking procedure can resolve the issue of low observability.

Due to the high nonlinearity of the bearing-only measurements, a 2-D BOT is difficult in the context of underwater target tracking [15]. To address this problem, a few nonlinear tracking techniques have been proposed for passive underwater target tracking scenarios. Among the techniques that ensure reliable tracking of an uncooperative underwater target, nonlinear Bayesian estimating techniques are the most popular due to their robust and accurate performance. By assuming that the disturbance caused by the environment has a Gaussian stochastic process, significant research accomplishments have been achieved by various researchers [16,17,18,19]. An extended Kalman filter (EKF)-based underwater target tracking algorithm was designed by Reference [16] to perform suboptimal estimations of an underwater target. Considering the linearization error introduced by the first-order Taylor series expansion, a group of determined sigma points was utilized by Reference [17] to linearize the nonlinear measurement equations in target tracking and thus to theoretically enhance the tracking accuracy of the second-order Taylor series expansion. Similarly, to enhance tracking accuracy, more complex tracking algorithms based on the cubature Kalman filter (CKF) and sigma-point Kalman filter with interpolation were proposed by References [18,19], respectively. Unlike the abovementioned studies, which utilized linearization techniques or determined sigma points to linearize the nonlinear system models, a particle filter (PF)-based estimating scheme using arbitrary “particles” to reform the nonlinear tracking system and to track the target was proposed by Reference [20]. By utilizing the Monte Carlo method to generate particles with an arbitrary distribution, the PF-based tracking algorithms can accurately estimate the distribution of the uncertainties of the tracked states. By generating many particles during the tracking procedure, the PF-based tracking algorithms can theoretically achieve accurate tracking results in any nonlinear tracking systems with any distributions of the uncertainties. However, the PF-based tracking algorithms have a significant computational burden, particularly when the system is highly nonlinear. As a result, the applications of PF-based tracking techniques are limited. 

Considering the linearization errors of nonlinear tracking algorithms and the significant computational load of PF-based tracking techniques, novel tracking mechanisms must be developed. In particular, new approaches are required for the scenario of bearing-only underwater uncooperative target tracking, in which the measurements are strongly nonlinear, but the kinematics is linear when the target operates in constant velocity (CV) mode. Rather than linearizing the system model to address the linearization error, a novel recursive tracking scheme, named the two-step filter, was developed by References [21,22]. By setting a nonlinear projection of the states to be tracked and the nonlinear measurements into a new extended space, the nonlinear measurement model can be transformed into a linear model in the first-step extended state estimation [21]. Then, by taking the nonlinear projection as a function of the original states, and the estimation bias from the first step, the original states can be extracted from the extended state via a recursive Newton iteration technique. The two-step filter has a superior performance when applied to nonlinear measurements because it eliminates the nonlinearities in the measurement update procedure, particularly when the measurements are abundant but the process model is simple [22]. In addition, based on the prototype designed by References [21,22], some modifications of the two-step filter have been made by several researchers. Reference [23] modified the time update procedure of the traditional two-step filter to ensure its robustness. This work was extended by Reference [24], in which a more comprehensive and complete time update method for the traditional two-step filter was proposed. Reference [25] proposed an adaptive two-step filter based on the modified Sage-Husa technique in combination with the multiple model technique to estimate the measurement noise online and thus to achieve higher tracking accuracy. In addition to theoretical studies, References [26,27] implemented the two-step filtering technique in applications of satellite attitude estimation and aerial target tracking. The studies verified the performance of the two-step filter and demonstrated its superior characteristics as an alternative solution to the various Bayesian nonlinear filters (i.e., EKF, UKF, PF, etc.). However, few studies can be found in which this approach has been implemented in underwater uncooperative target tracking despite the fact that the CV and nonlinear bearing-only measurement models represent a perfect tracking prototype for the two-step filter. In addition, modifications implemented by former researchers are not perfect in the underwater target tracking scenario because the uncertain underwater environment not only influences the measurements but also generates uncertainties in the model kinematics. Furthermore, the measurement update procedure has been paid little attention in previous research. As a result, few modifications have been made, leading to an incomplete theory of two-step tracking. 

This study aimed to robustly and accurately implement passive tracking of an underwater uncooperative target under the conditions of uncertain underwater disturbances while taking the advantages and shortcomings of existing tracking techniques into consideration. For this purpose, a novel adaptive two-step tracking algorithm is proposed. Inspired by the accomplishments of References [23,25] in the research area of aerospace engineering, this study adopted a modified Sage-Husa technique for online estimation of both the process noise and measurement noise to mitigate the influences caused by underwater uncertain disturbances. In addition, considering the specific scenario of underwater uncooperative target tracking in which measurements are limited, a negative matrix modification method in conjunction with a regularized Newton-Gauss iteration technique is proposed for both the time update and the measurement update procedures to ensure greater robustness and accuracy of the adaptive two-step filter. The main contributions of this study are summarized as follows:

1. First, a modified Sage-Husa online noise estimator was developed to simultaneously estimate the uncertain process noise and measurement noise during the underwater uncooperative target tracking procedure;
2. Second, a negative matrix modification method was utilized in the first step of the ATSF to ensure the time update process was steady. In addition, a regularized Newton-Gauss iteration technique was used in the measurement updating procedure to increase the robustness of the numerical recursion operation;
3. Finally, an adaptive two-step filter (ATSF) that combines an online noise estimator and a robust numerical recursive technique was used to robustly and accurately track the underwater uncooperative target.

The remainder of this paper is organized as follows. In Section 2, the problem mentioned above is formulated by introducing the kinematics of the underwater uncooperative target and the bearing-only measurement model. In Section 3, the principle of nonlinear least-squares estimation and the scheme of the traditional two-step filter for passive underwater uncooperative target tracking are reviewed. The modified Sage-Husa online noise estimator, the negative matrix modification method, the regularized Newton-Gauss iteration technique, and the adaptive two-step filter (ATSF) for underwater uncooperative target tracking are proposed in Section 4. Section 5 and Section 6 present the comprehensive simulation results and a discussion, respectively, to verify the effectiveness of the designed algorithm. Finally, in Section 7, the conclusions are drawn.

## 2. Problem Formulation

The 2-D bearing-only underwater uncooperative target tracking problem is formulated in this section by modeling the linear CV kinematics of the underwater uncooperative target and the nonlinear bearing-only measurements from two observers. The following section provides the details of the models.

### 2.1. Linear Kinematic Model of the Underwater Uncooperative Target

We assume that the underwater uncooperative target performs under constant velocity (CV) mode because the underwater uncooperative target is usually not maneuvering to save energy and to remain concealed. As the depth of the underwater uncooperative target is usually significantly smaller than the tracking range and the underwater target is usually maintained at a constant depth, the tracking procedure only focuses on the 2-D tracking of the XOY plane. Consider that $\left[\begin{array}{cc} x & y \end{array}\right]$ represents the current 2-D location of the underwater target and $\left[\begin{array}{cc} v_{x} & v_{y} \end{array}\right]$ represents the 2-D velocities. According to Reference [9], the kinematics model of the underwater uncooperative target can be represented as the following discrete formation:

(1) $$X_{k}=\Phi_{k/k-1}X_{k-1}+W_{k}$$

where $X_{k}={\left[\begin{array}{cccc} x_{k} & y_{k} & v_{xk} & v_{yk} \end{array}\right]}^{T}$ is the system state at tracking time k and where $W_{k}$ is the Gaussian process noise caused by the unknown underwater environment with first- and second-order moments, q and Q, respectively. $\Phi_{k/k-1}$ is the state transition matrix and can be represented as the following matrix if the target is in the CV operation mode:

(2) $$\Phi_{k/k-1}=\left[\begin{array}{cccc} 1 & 0 & T & 0\\ 0 & 1 & 0 & T\\ 0 & 0 & 1 & 0\\ 0 & 0 & 0 & 1 \end{array}\right]$$

### 2.2. Nonlinear Bearing-Only Measurement Model by Two Observers

**Figure 1:**
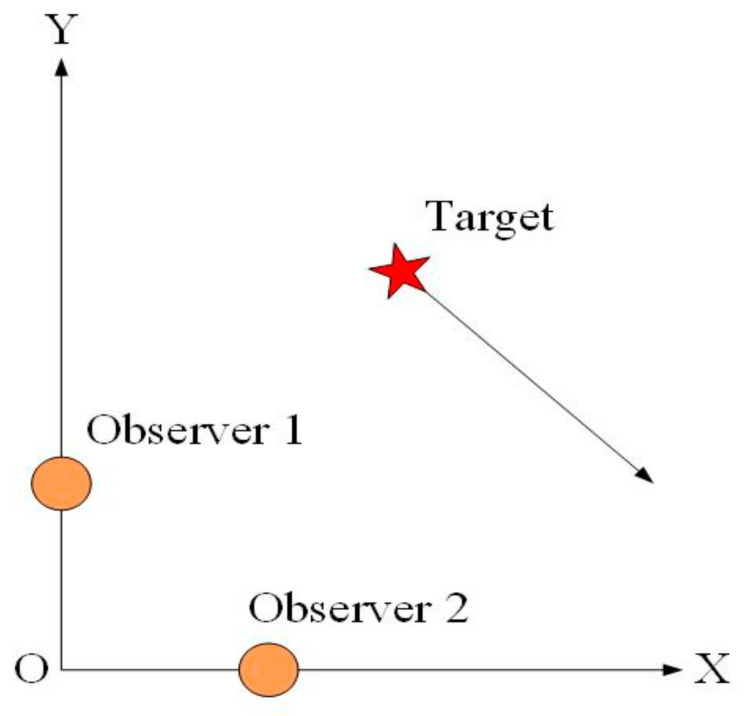
The configuration of the distributed bearing-only measurement system.

As single bearing-only measurements cannot ensure that the 2-D underwater target tracking problem is fully observable, this paper considers that a distributed bearing-only measurement system simultaneously provides two independent bearing measurements. The configuration of the two observers and the uncooperative underwater target is shown in Figure 1. 

**Figure 2:**
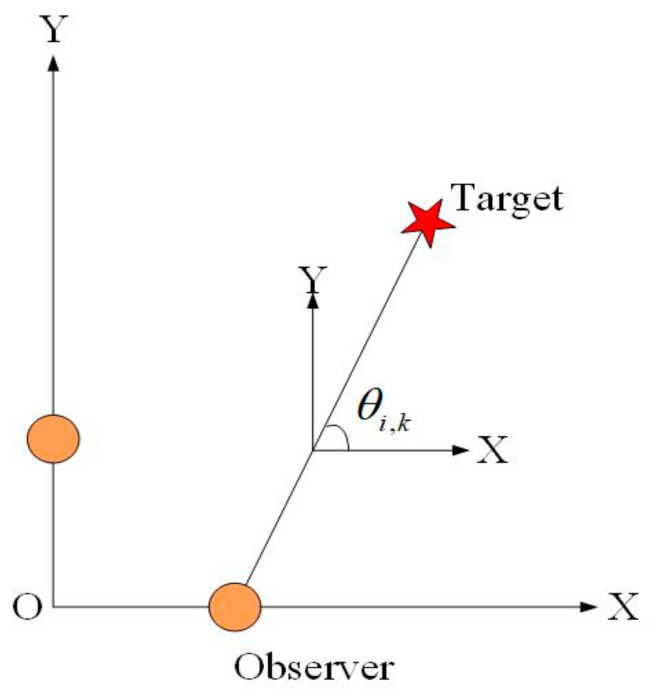
Measurement of the target by the ith observer in the XOY plane.

Considering that the two-independent bearing-only measurements are utilized at every tracking step, the measurements model can be expressed as follows:

(3) $$\begin{array}{ll} z_{k} & =\left[\begin{array}{c} \theta_{1,k}\\ \theta_{2,k} \end{array}\right]+V_{k}=h(X_{k})+V_{k}\\ & =\left[\begin{array}{c} \arctan\frac{y_{k}-y_{1,k}}{x_{k}-x_{1,k}}\\ \arctan\frac{y_{k}-y_{2,k}}{x_{k}-x_{2,k}} \end{array}\right]+\left[\begin{array}{c} V_{1,k}\\ V_{2,k} \end{array}\right] \end{array}$$

where $\left(x_{i,k}y_{i,k})(i=1,2\right)$ is the location of the ith observer at tracking time k of the XOY plane. $\theta_{i,k}(i=1,2)$ represents the bearing-only measurements measured by the ith observer at tracking time k. Similar to the consideration of $W_{k}$ in the process model, $V_{i,k}(i=1,2)$ is also modeled as the Gaussian measurement noise caused by the underwater environment with first- and second-order moments, $r_{k}$ and $R_{k}$, respectively. In this paper, we assume that both observers have the same stochastic process of the measurement noise. The overview of the target to the ith observer in the XOY plane is shown in Figure 2.

## 3. Nonlinear Least-Squares Estimation and the Traditional Two-Step Filter for Bearing-Only Underwater Uncooperative Target Tracking

### 3.1. Nonlinear Least-Squares Estimation for Underwater Uncooperative Target Tracking

From Equations (1) and (3), it is clear that the bearing-only underwater uncooperative target tracking system is nonlinear with a linear process equation and a nonlinear measurement equation. Thus, according to Reference [21], the principle of the nonlinear least-squares estimation of the bearing-only underwater target tracking problem can be restricted to minimize the following quadratic cost function subject to the system model formed by Equations (1) and (3):

(4) $$J=\frac{1}{2}{\left(X_{0}-{\bar{X}}_{0}\right)}^{T}P_{X_{0}}^{-1}\left(X_{0}-{\bar{X}}_{0}\right)+\frac{1}{2}\sum_{k=0}^{N}W_{k}^{T}Q_{k}^{-1}W_{k}+\frac{1}{2}\sum_{k=0}^{N}{\left[z_{k}-h\left(X_{k}\right)\right]}^{T}R_{k}^{-1}\left[z_{k}-h\left(X_{k}\right)\right]$$

where $X_{0}$ and ${\bar{X}}_{0}$ are the initial value and initial expectation of the states of the underwater uncooperative target, respectively. $P_{X_{0}}^{}$ is the initial covariance matrix, and the other parameters have the same definitions as those of Section 2. N is the total number of discrete tracking steps.

From Equation (4), it can be found that the goals of the bearing-only tracking procedure are to eliminate the initial errors between the real initial states and the initial estimates (the first term of Equation (4)), to mitigate the influence of the disturbance caused by the uncertain underwater environment (the second term of Equation (4)), and to minimize the measurement noise (the third term of Equation (4)). Hence, to obtain a robust and accurate tracking process, the initial errors and the uncertain disturbances of both the process model and measurement model should be taken into consideration. 

To minimize the quadratic cost function depicted by Equation (4) for the bearing-only underwater uncooperative target tracking scenario, one can adjoin the system model and the cost function using Lagrange multipliers [21]. It is clear that the tracking algorithm is a KF approach if the measurements model is linear and a global optimal can be ensured. However, due to the high nonlinearity in Equation (3), it is impossible to achieve globally optimal tracking results in the problem of bearing-only underwater target tracking, and every linearization of Equation (3) can be regarded as introducing linearization errors to the optimal estimation problem. In particular, from Equations (3) and (4), the errors existing in the measurements may lead to low estimation accuracies because the measurements contain the most information, from theoretical models and practical applications, to tune the tracking system.

### 3.2. Traditional Two-Step Filter for Bearing-Only Underwater Uncooperative Tracking

Considering the abovementioned issues, the traditional two-step filter was proposed by References [21,22] without a linearization procedure in the measurements model to better utilize the measurements and thus to improve the tracking results. Before implementing the traditional two-step filter in the bearing-only underwater uncooperative tracking problem, the principle of the two-step filter should be reviewed.

Considering a linear measurement model, Equation (4) can be represented as follows:

(5) $$J=\frac{1}{2}{\left(X_{0}-{\bar{X}}_{0}\right)}^{T}P_{X_{0}}^{-1}\left(X_{0}-{\bar{X}}_{0}\right)+\frac{1}{2}\sum_{k=0}^{N}W_{k}^{T}Q_{k}^{-1}W_{k}+\frac{1}{2}\sum_{k=0}^{N}{\left[z_{k}-H_{k}X_{k}\right]}^{T}R_{k}^{-1}\left[z_{k}-H_{k}X_{k}\right]$$

where the measurement model can be represented as follows:

(6) $$z_{k}=H_{k}X_{k}+V_{k}$$

with a measurement noise $V_{k}$, which has the same definition as that in Section 2.

According to the optimal control theory, the least-squares estimation of a linear system can be derived using the KF method and the tracking procedure is stable when the system is observable [26]. Consequently, the quadratic cost function can be simplified to the following:

(7) $$J=\frac{1}{2}\sum_{k=0}^{N}{\left[z_{k}-H_{k}X_{k}\right]}^{T}R_{k}^{-1}\left[z_{k}-H_{k}X_{k}\right]$$

Equation (7) shows that the optimal tracking results can be obtained by minimizing the measurement quadratic cost function if the system is linear, and the initial error and the process disturbances can be eliminated during the estimation process. Hence, to linearize the measurement model in the bearing-only underwater target tracking system represented by Equation (3), the extended state $y_{k}$ is first introduced as follows:

(8) $$y_{k}=f_{y}\left(X_{k}\right)=\left(\begin{array}{c} X_{k}\\ h\left(X_{k}\right) \end{array}\right)$$

Depending on the extended state $y_{k}$, the measurement model depicted by Equation (3) can be rewritten as follows:

(9) $$z_{k}=Y_{k}y_{k}+V_{k}$$

where

(10) $$Y_{k}=\left[\begin{array}{c} \begin{array}{cccccc} 0 & 0 & 0 & 0 & 1 & 0 \end{array}\\ \begin{array}{cccccc} 0 & 0 & 0 & 0 & 0 & 1 \end{array} \end{array}\right]$$

is the extended measurement matrix.

From Equations (8) and (9), it can be found that the nonlinear bearing-only underwater uncooperative target tracking system formed by Equations (1) and (3) has been transformed to linear expressions by the extended state $y_{k}$ with the measurement matrix $Y_{k}$. Therefore, the quadratic cost function to be minimized by the extended state can be represented as minimizing the following linearized quadratic cost function:

(11) $$J=\frac{1}{2}\sum_{k=0}^{N}{\left[Z_{k}-Y_{k}y_{k}\right]}^{T}R_{Vk}^{-1}\left[z_{k}-Y_{k}y_{k}\right]$$

where $R_{Vk}^{}$ is the covariance matrix of the extended measurement noise.

Thus, from the optimal control theory [28], the nonlinear bearing-only underwater uncooperative target tracking problem can be first solved using the KF method under the extended states to obtain its optimal solution. According to Reference [28], the extended state $y_{k}$ can be computed recursively using the KF method under the system model formed by Equations (1) and (3) via the following time update and measurement update steps:

1. Time update:
  (12) $$X_{k/k-1}=\Phi_{k}X_{k-1/k-1}$$
  (13) $$P_{k/k-1}=\Phi_{k}P_{k-1/k-1}\Phi_{k}^{T}+Q_{k}$$
  (14) $$y_{k/k-1}=y_{k-1/k-1}+f_{y}\left(X_{k/k-1}\right)-f_{y}\left(X_{k-1/k-1}\right)$$
  (15) $$P_{y,k/k-1}=P_{y,k-1/k-1}+F_{k/k-1}P_{k/k-1}F_{k/k-1}^{T}-F_{k-1/k-1}P_{k-1/k-1}F_{k-1/k-1}^{T}$$
2. Measurement update:
  (16) $$K_{k}=P_{y,k/k-1}Y_{k}^{T}{\left(Y_{k}P_{y,k/k-1}Y_{k}^{T}+R_{k}\right)}^{-1}$$
  (17) $$y_{k/k}=y_{k/k-1}+K_{k}\left(z_{k}-Y_{k}y_{k/k-1}\right)$$
  (18) $$P_{y,k/k}=\left(I-K_{k}Y_{k}\right)P_{y,k-1}{\left(I-K_{k}Y_{k}\right)}^{T}+K_{k}R_{k}K_{k}^{T}$$
  where $F_{k/k-1}=\frac{\partial f_{y}\left(X_{k/k-1}\right)}{\partial X_{k/k-1}}$ and $F_{k-1/k-1}=\frac{\partial f_{y}\left(X_{k-1/k-1}\right)}{\partial X_{k-1/k-1}}$ are the Jacobian matrices of the extended state at $X_{k/k-1}$ and $X_{k-1/k-1}$, respectively.

Equations (12) to (18) recursively yield the optimal estimation of the extended sate $y_{k}$ and its covariance matrix $P_{yk}$. This optimal extended state estimation process is called the first-step extended state estimation. As the extended state linearizes the passive underwater uncooperative target tracking system, the estimation results are optimal and are not affected by the initial errors when the system is fully observable. This is particularly the case when the target is uncooperative and prior information is too limited to allow for an accurate initial guess. In addition, it is clear that the nonlinearities are removed from the measurement process, which can ensure the tracking procedure makes full use of the measurements to enhance the estimation accuracy. Instead, the nonlinearities of the measurement models are transformed to the time update process with original states at different tracking steps. This adds more information to the nonlinear measurement model compared to the traditional linearization methods, which only linearize the nonlinear measurement model at the current state. Therefore, by adopting the extended state in the first step, the nonlinearity of the passive underwater uncooperative target tracking system decreases and the estimation accuracy is enhanced.

Then, considering the original state $X_{k}$ defined in Section 2.1, and the extended state $y_{k}$ and the estimation error in the first step, the following recursive equation can be obtained:

(19) $$y_{k/k}=f_{y}\left(X_{k}\right)+V_{y,k}$$

where $V_{y,k}$ is the estimation error of the first-step extended state. 

According to Reference [28], the original state can be extracted from the extended state by minimizing the following quadratic cost function:

(20) $$J_{y}=\frac{1}{2}{\left(y_{k/k}-f_{y}\left(X_{k}\right)\right)}^{T}P_{y,k/k}^{-1}\left(y_{k/k}-f_{y}\left(X_{k}\right)\right)$$

Utilizing the Newton-Gauss iteration technique, the original state can be computed recursively as follows:

(21) $$X_{k}^{\left(i+1\right)}=X_{k}^{\left(i\right)}-{\left(G_{k}^{\left(i\right)}\right)}^{-1}{\left(g_{k}^{\left(i\right)}\right)}^{T}$$

where

(22) $${\left.{\left.G_{k}^{\left(i\right)}≐\frac{\partial f_{y}}{\partial X_{k}^{}}\right|}_{X_{k}^{\left(i\right)}}P_{y,k/k}^{-1}\frac{\partial f_{y}}{\partial X_{k}^{}}\right|}_{X_{k}^{\left(i\right)}}$$

and

(23) $$g_{k}^{\left(i\right)}={\left.\frac{\partial J_{y}}{\partial X_{k}^{\left(i\right)}}\right|}_{X_{k}^{(i)}}=-{\left.{\left[y_{k/k}-f_{y}\left(X_{k}^{\left(i\right)}\right)\right]}^{T}P_{y,k/k}^{-1}\frac{\partial f}{\partial X_{k}^{}}\right|}_{X_{k}^{\left(i\right)}}$$

Equations (21) to (23) form the second-step original state estimation scheme. From the Newton–Gauss iteration technique, the original state $X_{k}$ can be extracted from the extended state $y_{k}$ that was recursively estimated from the first step.

Therefore, by utilizing the traditional two-step filter technique and by considering the passive underwater uncooperative target tracking system depicted by Equations (1) and (3), the traditional two-step filter for the bearing-only underwater uncooperative tracking algorithm can be summarized in Algorithm 1 as follows:
**Algorithm 1: Traditional two-step filter for bearing-only underwater uncooperative tracking.**1:Considering the system model depicted by Equations (1) and (3), initialize the state $X_{0}$ and the covariance matrix $P_{0}$;2:Set up the initial extended sate $y_{0}$ and extended covariance matrix $P_{y0}$ according to Equation (8);
3:Estimate the extended state $y_{k}$ and the extended covariance matrix $P_{y,k}$ at the kth tracking step by the first-step estimation depicted by Equations (12) to (18);4:Extract the original state $X_{k}$ from the extended state $y_{k}$ by the second-step estimation depicted by Equations (21) to (23);5:Calculate the iteration difference $\delta =\left\|X_{k}^{i+1}-X_{k}^{i}\right\|$, and check whether δ is less than the preset threshold for stopping iteration. If δ is larger than the preset threshold, the iteration for the second step continues; otherwise stop the iteration of the second step;
6:Time propagation to run the whole algorithm at tracking time k+1. Then, jump to step (3).

## 4. Adaptive Two-Step Bearing-Only Underwater Uncooperative Target Tracking

From Algorithm 1 proposed in the former section, two main drawbacks exist in the traditional two-step filter. First, the traditional two-step filter assumes the process noise, and the measurement noise remain unchanged and are known as prior information during the first-step estimation. However, in a real bearing-only underwater uncooperative target tracking scenario, the process noise and the measurement noise are unknown and uncertain. As a result, online noise estimating techniques should be considered to increase the robustness and accuracy of the tracking procedure. In addition, both the first-step and second-step iteration processes have their own drawbacks. As described in References [23,24], Equation (14) in the time-update procedure of the first-step estimation sometimes becomes negative. To address this problem, References [23,24] provide several numerical and analytical solutions to avoid the negative time-updated extended covariance matrix. Then, in the second step, the original state $X_{k}$ is extracted recursively from the estimated extended state $y_{k}$ by the Newton-Gauss method. However, the iteration procedure sometimes diverges because, during the bearing-only underwater uncooperative target tracking procedure, the Hessian matrix becomes ill-conditioned. 

Considering the uncertainties in the tracking process, various kinds of adaptive tracking techniques have been developed to deal time different uncertain situations. References [29,30] considered the irregular sampling time during the measuring procedure, several robust parameter estimating algorithms are proposed. If the target is a far slow-moving object, the measurement intervals can be regarded as regular and adaptive tracking techniques dealing with the uncertain noise can be adopted to enhance the tracking accuracy. Inspired by the former research of References [23,24,25,27] and considering the application simplicity and the estimation performance, an adaptive two-step bearing-only underwater uncooperative target tracking filter (ATSF) is proposed in this paper. The proposed ATSF consists of three main parts, namely, online noise estimation, first-step negative matrix correction, and the second-step regularized Newton-Gauss iteration. The details are described in this section.

### 4.1. Modified Sage-Husa Online Noise Estimation

The Sage–Husa online noise estimator was first introduced by Reference [31] for linear system noise estimation. For the nonlinear tracking system depicted by Equations (1) and (3), the first and second momentum of the uncertain noise at tracking time k can be estimated online using the nonlinear Sage–Husa estimator as follows:

(24) $${\hat{q}}_{k}=\frac{1}{k}\sum_{j=1}^{k}\left({\hat{X}}_{j/j}-\Phi_{j/j-1}{\hat{X}}_{j-1/j-1}\right)$$

(25) $${\hat{Q}}_{k}=\frac{1}{k}\sum_{j=1}^{k}\left[\Delta X_{j}-q\right]{\left[\Delta X_{j}-q\right]}^{T}$$

(26) $${\hat{r}}_{k}=\frac{1}{k}\sum_{j=1}^{k}\left(z_{j}-\left({\hat{X}}_{j/j}\right)\right)$$

(27) $${\hat{R}}_{k}=\frac{1}{k}\sum_{j=1}^{k}\left[\Delta z_{j}-r\right]{\left[\Delta z_{j}-r\right]}^{T}$$

where q and r are the first-order momentum of the process noise and measurement noise, respectively; $\Phi_{j/j-1}$ is the transfer matrix from tracking time j−1 to j; and $\Delta X_{j}$ and $\Delta z_{j}$ are represented as follows:

(28) $$\Delta X_{j}={\hat{X}}_{j/j}-\Phi_{j/j-1}{\hat{X}}_{j/j-1}$$

(29) $$\Delta Z_{j}=Z_{j}-h\left({\hat{X}}_{j/j-1}\right)$$

From Equations (24) to (29), it can be found that the classic nonlinear Sage–Husa online noise estimator must utilize all of the smooth values of the state within a certain tracking period; as a result, the total process is hard to compute. According to Reference [20], the recursive suboptimal representation of the nonlinear Sage–Husa online noise estimator can be represented as follows:

(30) $${\hat{q}}_{k}=\left(1-\frac{1}{k}\right){\hat{q}}_{k-1}+\frac{1}{k}\Delta X_{k}$$

(31) $${\hat{r}}_{k}=\left(1-\frac{1}{k}\right){\hat{r}}_{k-1}+\frac{1}{k}\Delta z_{k}$$

(32) $${\hat{Q}}_{k}=\left(1-\frac{1}{k}\right){\hat{Q}}_{k-1}+\frac{1}{k}\left(K_{k}\varepsilon_{k}\varepsilon_{k}^{T}K_{k}^{T}+P_{k/k}-\Phi_{k-1}P_{k-1/k-1}\Phi_{k-1}^{T}\right)$$

(33) $${\hat{R}}_{k}={\hat{R}}_{k-1}+\frac{1}{k}\left(\varepsilon_{k}\varepsilon^{T}{}_{k}-P_{zz}{}_{,k/k-1}\right)$$

where $K_{k}$ is the filter gain by a designed tracking algorithm, $P_{k/k}$ is the covariance of the estimated state at time k, and $\varepsilon_{k}$ is the innovation represented as the following equation:

(34) $$\varepsilon_{k}=z_{k}-h\left({\hat{X}}_{k/k-1}\right)-r_{k}$$

The representation of $P_{zz}{}_{,k/k-1}$ is dependent on different nonlinear tracking algorithms.

For the uncertain noise, the most recent measurement should be given more attentions than the historical data. Therefore, the fading factor $d_{k}$ at tracking time k is introduced to Equations (30) to (33) as follows:

(35) $${\hat{q}}_{k}=\left(1-d_{k}\right){\hat{q}}_{k-1}+d_{k}\Delta X_{k}$$

(36) $${\hat{r}}_{k}=\left(1-d_{k}\right){\hat{r}}_{k-1}+d_{k}\Delta z_{k}$$

(37) $${\hat{Q}}_{k}=\left(1-d_{k}\right){\hat{Q}}_{k-1}+d_{k}\left(K_{k}\varepsilon_{k}\varepsilon_{k}^{T}K_{k}^{T}+P_{k/k}-\Phi_{k-1}P_{k-1/k-1}\Phi_{k-1}^{T}\right)$$

(38) $${\hat{R}}_{k}=\left(1-d_{k}\right){\hat{R}}_{k-1}+d_{k}\left(\varepsilon_{k}\varepsilon^{T}{}_{k}-P_{zz}{}_{,k/k-1}\right)$$

with

(39) $$d_{k}=\frac{1-b}{1-b^{k}}$$

It can be found in Equation (39) that, when the index b is close to 1, the noise calculated by Equations (35) to (38) focuses on the measurements from the total tracking period. On the contrary, if b is close to 0, the estimated noise is more focused on the current time innovation. Consequently, by tuning the value of index b, the fading factor $d_{k}$ changes dynamically to affect the online noise estimation performance. By combining Equations (35) to (39), we obtain the modified Sage–Husa online noise estimator to address the uncertain characteristic of the process and measurement noise during the tracking procedure.

### 4.2. First-Step Negative Matrix Correction

In the first-step time update procedure of the traditional two-step filter described as Equation (15), the matrix subtraction operation sometimes results in an ill-conditioned $P_{y,k/k-1}$, as discovered by References [23,24]. Thus, to ensure the positive characteristic of the computed $P_{y,k/k-1}$, a small positive scalar ε is introduced to modify the original, as proposed by Reference [23] as the following:

(40) $$P_{y,k/k-1}=P_{y,k-1/k-1}+F_{k/k-1}P_{k/k-1}F_{k/k-1}^{T}-F_{k-1/k-1}P_{k-1/k-1}F_{k-1/k-1}^{T}+\varepsilon I$$

Although References [24,26] utilized a more comprehensive analytical expression to calculate the extended state and its covariance matrix in the first-step time updating step, the continuous differential computation introduces a significant amount of computational complexity. Therefore, we utilize the regularization technique to make $P_{y,k/k-1}$ positive.

### 4.3. Second-Step Regularized Newton-Gauss Iteration 

From Equation (21), the Hessian matrix is inverted during the iteration process. However, this inversion diverges when the values of components of the Hessian matrix $G_{k}^{\left(i\right)}$ are nearly zero, particularly when the target is uncooperative and few measurements can be obtained. Therefore, to ensure the robustness of tracking, a regularization technique is adopted. The implementation of the regularization process in Equation (21) can be represented as the following equation:

(41) $$X_{k}^{\left(i+1\right)}=X_{k}^{\left(i\right)}-{\left(G_{k}^{\left(i\right)}+\lambda I\right)}^{-1}{\left(g_{k}^{\left(i\right)}\right)}^{T}$$

where λ is the coefficient of the regularization process and the other matrixes have the same definitions as previously mentioned in Section 3.

From Equation (41), by setting a specific small value λ, the inversion computation can avoid the ill-conditioned situation and thus ensures a stable second-step iteration while extracting the original state $X_{k}^{}$ from the extended state $y_{k}$.

### 4.4. Adaptive Two-Step Filter for Bearing-Only Underwater Uncooperative Target Tracking

From Equations (37), (38), (40), and (41), in conjunction with the traditional two-step filter described in Section 3, the adaptive two-step filter (ATSF) for passive underwater uncooperative target tracking can be summarized as Algorithm 2, as follows:
**Algorithm 2: Adaptive two-step filter for bearing-only underwater uncooperative tracking.**1:Considering the system model depicted by Equations (1) and (3), initialize the state $X_{0}$ and the covariance matrix $P_{0}$;2:Set up the initial extended sate $y_{0}$ and extended covariance matrix $P_{y0}$ according to Equation (8);3:Estimate the process noise and measurement noise online by Equations (37) and (38);4:Estimate the extended state $y_{k}$ and the extended covariance matrix $P_{y,k}$ at the kth tracking step by the first-step estimation depicted by Equations (12) to (14), Equation (40), and Equations (16) to (18);5:Extract the original state $X_{k}$ from the extended state $y_{k}$ by the second-step estimation depicted by Equations (41), (22), and (23);
6:Calculate the iteration difference $\delta =\left\|X_{k}^{i+1}-X_{k}^{i}\right\|$, and check whether δ is less than the preset threshold for stopping the iteration. If δ is larger than the preset threshold, the iteration for the second step continues; otherwise stop the iteration of the second step;
7:Time propagation to run the whole algorithm at tracking time k+1. Then, jump to step (3).

Compared to Algorithm 1 proposed in Section 3, it is clear that the modified step (3) in Algorithm 2 enables the online noise estimation process to increase the accuracy of the tracking procedure. In addition, the modified steps (4) and (5) in Algorithm 2 ensure that the two-step filter has a robust estimation performance. Hence, the proposed ATSF can increase the robustness and accuracy of the passive underwater uncooperative target tracking process. 

## 5. Simulations and Discussions

### 5.1. Simulation Scenario

As presented in this section, comprehensive simulations were undertaken to verify the proposed bearing-only 2-D underwater uncooperative target passive tracking algorithm.

In addition, by setting the reference coordination, the coordinates of each observer and the target are represented in the reference coordination. The detailed configuration of the observers is presented in Table 1. In this paper, we assume that both observers have the same sensing and communication range.

In the simulations, the underwater target was assumed to perform in CV motion mode with an actual initial state as $X_{0}=\left[\begin{array}{cccc} 2000 & 2000 & 5 & -4 \end{array}\right]$ and a constant velocity $[\begin{array}{cc} v_{x} & v_{y} \end{array}]=\left[\begin{array}{cc} 5 & -4 \end{array}\right]m/s$ from tracking time 0 to 200 s. In addition, to verify the tracking performance of the proposed ATSF, different initial guesses are presented in Table 2 with the same covariance matrix of $P_{0}=diag(\begin{array}{cccc} 100,000 & 10,000 & 1000 & 1000 \end{array})$.

In addition, the uncertain process noise was modeled as a combination of the certain noise and an uncertain adding term. For the tracking process, the stochastic process of the certain noise is known a priori and the uncertain adding noise is unknown. The process noise is modeled as the following equations:

(42) $$W_{k}={\left[\begin{array}{cccc} 0 & 0 & w_{vx} & w_{vy} \end{array}\right]}^{T}$$

with

(43) $$w_{vi}={\bar{w}}_{vi}+N_{wi}\left(i=x,y\right)$$

where ${\bar{w}}_{vi}(i=x,y)$ and $N_{wi}(i=x,y)$ are the zero-mean Gaussian white noise, with expectations of the variance matrix of $E\left({\bar{w}}_{vi,1}{\bar{w}}_{vi,1}^{T}\right)=0.01$ and $E\left(N_{wi}N_{wi}^{T}\right)=0.005$, respectively.

Furthermore, it was assumed that both observers have the same stochastic process of the measurement noise and can be modeled as follows:

(44) $$V_{i,k}={\left[v_{\theta_{i}}\right]}^{T}\left(i=1,2\right)$$

with

(45) $$v_{\theta_{i}}={\bar{v}}_{\theta_{i}}+N_{vi}\left(i=1,2\right)$$

where ${\bar{v}}_{\theta_{i}}$ and $N_{vi}(i=1,2)$ are the zero-mean Gaussian white noise, with expectations of the variance matrix of $E\left({\bar{v}}_{\theta_{i}}{\bar{v}}_{\theta_{i}}^{T}\right)=0.25$ and $E\left(N_{vi}N_{vi}^{T}\right)=0.05$, respectively.

All of the simulations presented in this paper were performed using MATLAB R2019b on a computer with a Microsoft Windows 10 system; the computer was configured with Intel (R) Core (TM) i7-9700k CPU @3.2 GHz. The simulation results are the average of 50 Monte Carlo experiments. The total simulation time was set as 200 s with a 1 s measurement interval. To evaluate the performance of the proposed algorithm, the root mean square error (RMSE) for locations, velocities, and the angular velocity of the underwater uncooperative target were used and can be represented as follows:

(46) $$\begin{array}{l} RMSE_{l}=\sqrt{\frac{1}{N}\sum_{i=1}^{N}\left(\Delta x_{k}^{2}+\Delta y_{k}^{2}\right)}\\ RMSE_{v}=\sqrt{\frac{1}{N}\sum_{i=1}^{N}\left(\Delta v_{xk}^{2}+\Delta v_{yk}^{2}\right)} \end{array}$$

with

(47) $$\begin{array}{l} \Delta x_{k}^{}=x_{k}-{\bar{x}}_{k}\\ \Delta y_{k}^{}=x_{k}-{\bar{y}}_{k} \end{array}$$

and

(48) $$\begin{array}{l} \Delta v_{xk}^{}=v_{xk}-{\bar{v}}_{xk}\\ \Delta v_{yk}^{}=v_{yk}-{\bar{v}}_{yk} \end{array}$$

where N is the total number of the Monte Carlo trials; $\left(\begin{array}{cc} x_{k} & y_{k} \end{array}\right)$ and $\left(\begin{array}{cc} {\bar{x}}_{k} & {\bar{y}}_{k} \end{array}\right)$ are the real and estimated locations of the underwater target, respectively; and $\left(\begin{array}{cc} v_{xk} & v_{yk} \end{array}\right)$ and $\left(\begin{array}{cc} {\bar{v}}_{xk} & {\bar{v}}_{yk} \end{array}\right)$ are the real and estimated velocities of the underwater target, respectively.

### 5.2. Simulation Results and Discussions

As presented in this subsection, the ATSF proposed in Section 4 was tested and compared to the traditional two-step filter and the extended Kalman filter used in Reference [16], under the same simulation environment for bearing-only underwater uncooperative target tracking scenario. The simulation results and discussions are described in detail in this subsection.

#### 5.2.1. Accuracy Analysis under Different Initial Errors

**Figure 3:**
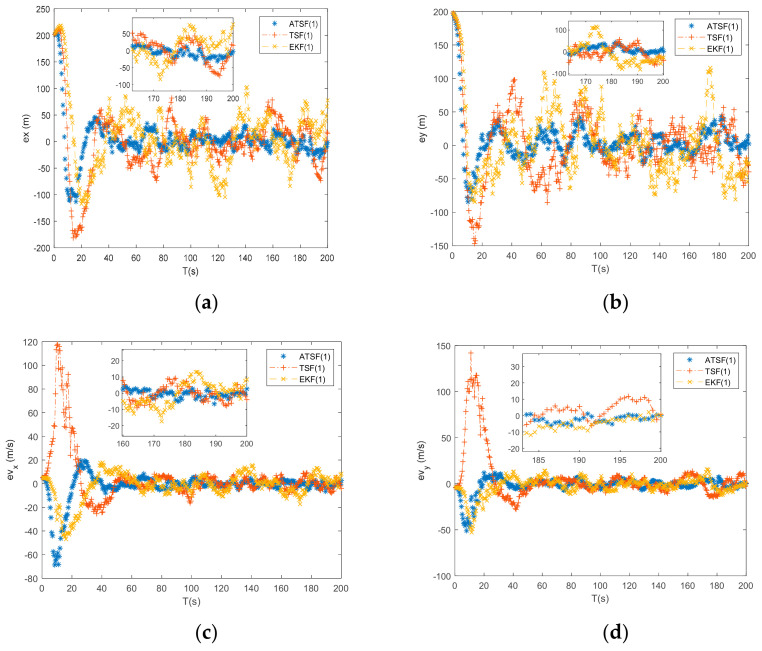
(**a**) Error of x by ATSF, TSF, and EKF under initial guess 1; (**b**) error of y by ATSF, TSF, and EKF under initial guess 1; (**c**) error of $v_{x}$ by ATSF, TSF, and EKF under initial guess 1; and (**d**) error of $v_{y}$ by ATSF, TSF, and EKF under initial guess 1.

**Figure 4:**
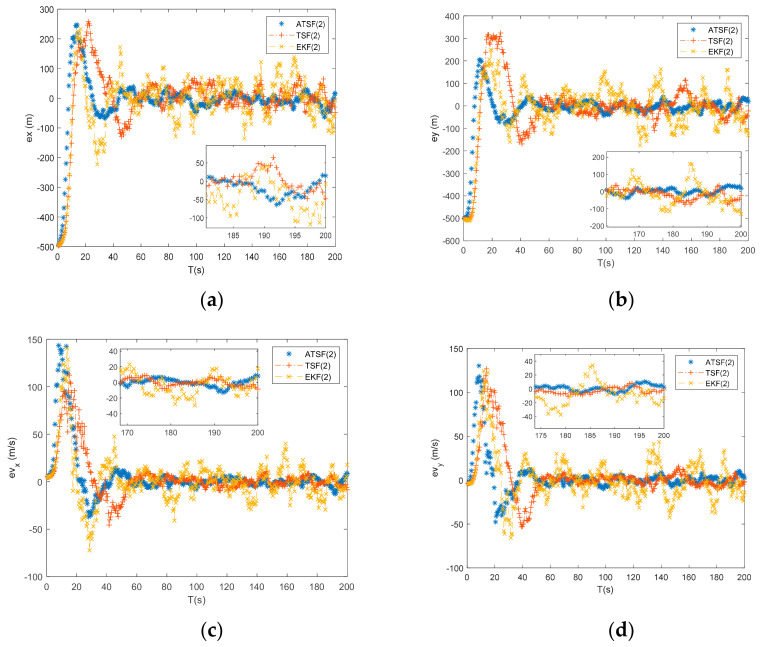
(**a**) Error of x by ATSF, TSF, and EKF under initial guess 2; (**b**) error of y by ATSF, TSF, and EKF under initial guess 2; (**c**) error of $v_{x}$ by ATSF, TSF, and EKF under initial guess 2; and (**d**) error of $v_{y}$ by ATSF, TSF, and EKF under initial guess 2.

**Figure 5:**
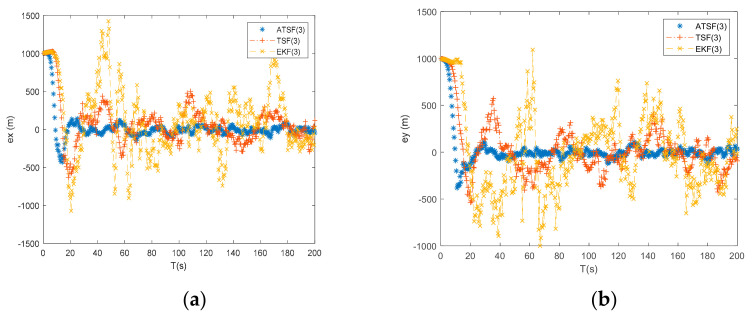

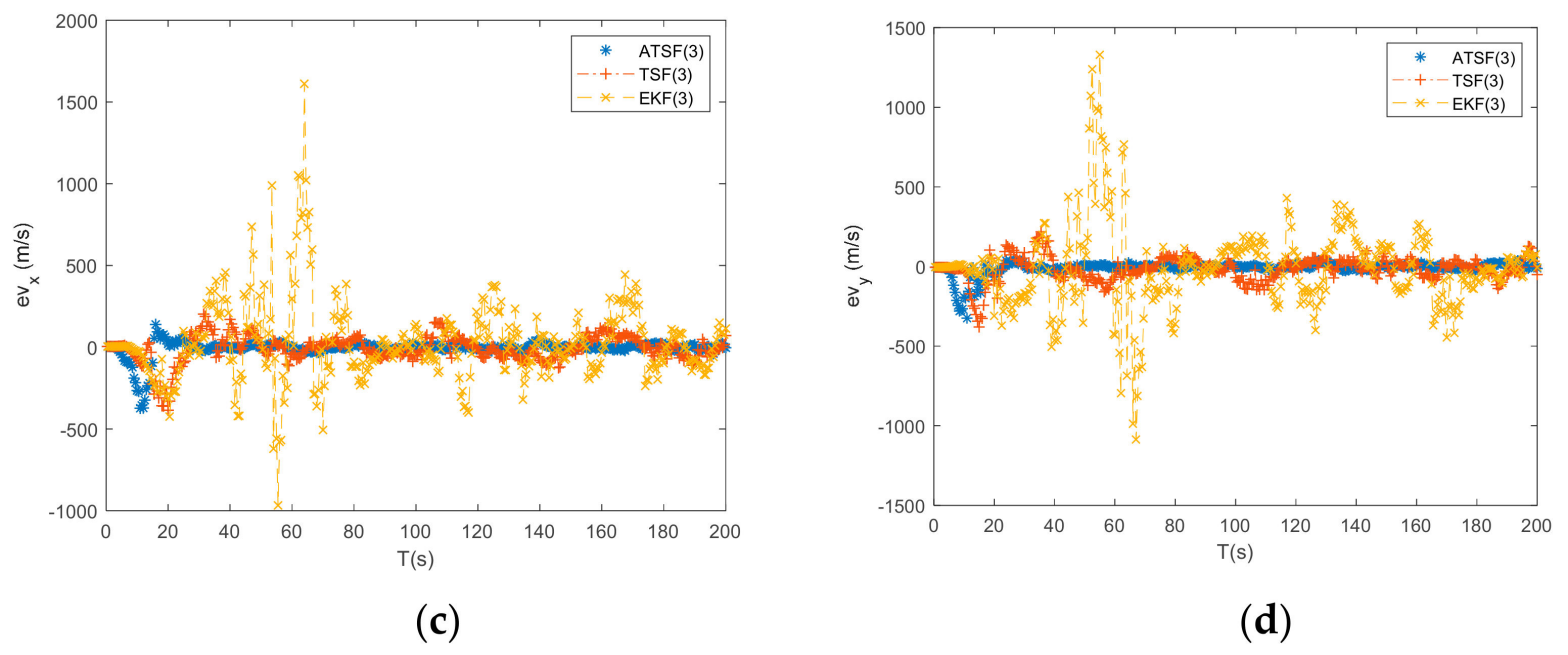
(**a**) Error of x by ATSF, TSF, and EKF under initial guess 3; (**b**) error of y by ATSF, TSF, and EKF under initial guess 3; (**c**) error of $v_{x}$ by ATSF, TSF, and EKF under initial guess 3; and (**d**) error of $v_{y}$ by ATSF, TSF, and EKF under initial guess 3.

According to the three different initial guesses presented in Table 2, the tracking results from the ATSF, the traditional two-step filter (TSF), and the EKF are shown in Figure 3, Figure 4 and Figure 5, with the calculated $RMSE_{l}$ and $RMSE_{l}$ shown in Table 3. ATSF (1) in Table 3 means the tracking results from the ATSF under the initial guess with number 1 and the other notations are defined similarly.

From Figure 3 and the tracking results with number 1 (under initial guess 1 from Table 2) shown in Table 3, it is clear that all three tracking algorithms can converge within the simulation time. From Table 3, the localization error between the ATSF and the EKF is 12.25 m and the velocity error is 1.73 m/s. In addition, the tracking accuracy of the ATSF is higher than that of the traditional two-step filter in terms of its online noise estimation and robust iteration characteristics. Although the ATSF has the highest tracking accuracy, the differences among the three tracking algorithms are minor, and the bias between the initial value of the target and the initial guesses of the tracking algorithm is small. Consequently, the linearization error has a limited influence during the tracking procedure.

From Figure 4 and the tracking results with number 2 (under initial guess 2 from Table 2) shown in Table 3, the differences between the ATSF and EKF are apparent. Due to a larger initial bias, the initialization error in the EKF significantly influences the final tracking accuracy, and the two-step tracking techniques can achieve substantially greater tracking accuracy by shifting the initialization error from the measurement-updating process to the time-updating process. In addition, from the tracking results, the ATSF can achieve a higher tracking accuracy than the traditional two-step filter since it can estimate the uncertain noise online to adjust its performance at each tracking step. From Figure 4, it can also be seen that the traditional two-step filter fluctuates during the tracking process compared to the proposed ATSF. This phenomenon occurs because the ATSF adopted the regularization technique to ensure the tracking procedure remains steady. As a result, when the initial error becomes large, the proposed ATSF has superior performance compared to the EKF and can achieve more stable and robust results than the traditional two-step filter.

From Figure 5 and the tracking results with number 3 (under initial guess 3 from Table 2) shown in Table 3, the proposed ATSF is the only method that can provide reliable tracking results. As the initial errors are relatively large, the linearization-based EKF diverged after several tracking steps. In addition, for the uncertain noise and ill-conditioned matrixes in the tracking procedure, the tracking accuracy of the traditional two-step filter is limited, although it can converge within the tracking period. However, the ATSF can achieve reliable and robust tracking under this circumstance due its online noise estimating ability and stable iteration characteristic. As a result, for the bearing-only underwater uncooperative target tracking scenario, the ATSF has superior tracking ability since a priori knowledge is unavailable for an underwater uncooperative target and a large initial bias exists.

#### 5.2.2. Computational Time Analysis under Different Thresholds δ during Regularized Newton-Gauss Iteration

From Section 3 and Section 4, it is clear that the threshold δ is the key parameter that affects the efficiency of the two-step tracking scheme. If δ is set to a smaller value, the total iteration process becomes relatively longer and the tracking accuracy becomes higher. To increase the applicability of the proposed algorithm, computational load analysis was undertaken, as presented in this subsection, to discover a balance between the computing time and the tracking accuracy of the proposed ATSF. 

**Figure 6:**
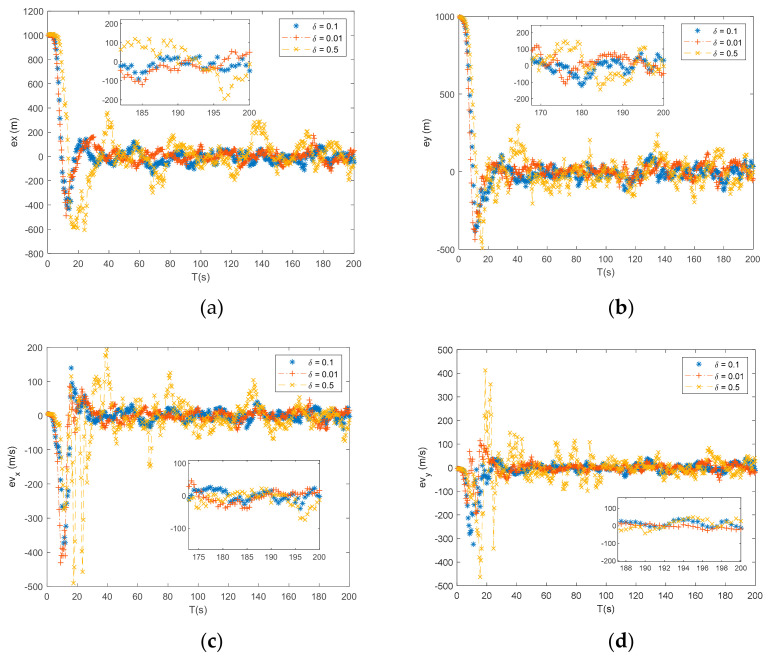
(**a**) Error of x by ATSF using different iteration thresholds; (**b**) error of y by ATSF using different iteration thresholds; (**c**) error of $v_{x}$ by ATSF and TSF using different iteration thresholds; and (**d**) error of $v_{y}$ by ATSF using different iteration thresholds.

Under the initial guess 3 from Table 2, the ATSF was implemented under three different thresholds $\delta_{i}(i=1,2,3)$, and the tracking results, tracking accuracy, and computational time are shown in Figure 6 and Table 4.

It can be found in Figure 6 and Table 4 that a smaller threshold can provide a relatively accurate tracking result. However, the computational time increases when a smaller threshold is adopted. From the tracking results of $\delta_{1}$ and $\delta_{2}$, the accuracy was significantly enhanced at the expense of additional computational time, which increased by 15.13 s. In addition, from the tracking results of $\delta_{1}$ and $\delta_{3}$, the tracking accuracy was slightly enhanced but the computational time increased by 193.7 s. The simulation results indicate that a suitable threshold should be selected to balance the tracking accuracy and the computational time. In our case, threshold $\delta_{1}$ is optimal.

## 6. Discussion

From the comprehensive simulation results and analysis, a brief summary can be made of the following empirical principles:

1. The proposed ATSF, the traditional two-step filter, and the EKF can obtain good tracking results when the initial errors are small. This phenomenon occurs because the linearization error is limited by small errors between the initial values of the target and the initial guesses of the tracking algorithms. However, when the initial errors become large, the tracking performances are different. The superior tracking performance of the two-step filtering scheme for both the ATSF and the traditional two-step filter shows their effectiveness.
2. As the ATSF is more robust and accurate due to the online noise estimation function and the regularization operation in the two-step filtering process, the ATSF can achieve more accurate tracking results when the online noise varies. In addition, the tracking performance of the ATSF is more stable than that of the traditional two-step filter because the regularization operations prevent the core matrixes from becoming ill-conditioned during the filtering procedure. On the contrary, when the noise is uncertain, the tracking procedure of the traditional two-step filter fluctuates and the tracking results have larger errors when compared to the ATSF.
3. Regarding the computational time, the computational loads for the proposed ATSF and the traditional two-step filter rely significantly on the second-step Newton-Gauss iterating operation. A smaller difference between the state at the ith and i+1th iteration steps results in an increase in computational time. Therefore, setting a rational iteration threshold is important to control the computational load of the two-step filtering techniques. In real applications, this threshold can be predetermined for a specific tracking system by empirical simulations.

## 7. Conclusions

The 2-D bearing-only tracking of an underwater uncooperative target with uncertain underwater disturbances was examined in this study. By adopting the modified Sage-Husa online noise estimator in conjunction with the regularization technique in the two-step filtering scheme, the proposed ATSF can obtain accurate and robust tracking results under the condition of uncertain disturbances. The simulation results verified the superior tracking ability of the proposed ATSF, and comprehensive discussions are presented to provide empirical insight for the implementation of the proposed ATSF in real bearing-only underwater uncooperative tracking scenarios. It is believed that the ATSF proposed by this paper has significant potential in real-time 2-D bearing-only underwater target tracking missions. 

## Figures and Tables

**Table 1:** Detailed configuration of the observers.

Observer Number	Coordinate
1	$\left(x_{1},y_{1}\right)\;=\;\left(0,600\right)$
2	$\left(x_{2},y_{2}\right)\;=\;\left(600,0\right)$

**Table 2:** Different initial guesses.

Number	Initial Guesses
1	${\hat{X}}_{0}\;=\;\left[\begin{array}{cccc} 1800 & 1800 & 0 & 0 \end{array}\right]$
2	${\hat{X}}_{0}\;=\;\left[\begin{array}{cccc} 2500 & 2500 & 0 & 0 \end{array}\right]$
3	${\hat{X}}_{0}\;=\;\left[\begin{array}{cccc} 1000 & 1000 & 0 & 0 \end{array}\right]$

**Table 3:** The $RMSE_{l}$ and $RMSE_{l}$ for the ATSF, the traditional two-step filter (TSF), and the EKF for different initial guesses.

Tracking Algorithm	$RMSE_{l}\;(m)$	$RMSE_{v}\;(m/s)$
ATSF (1)	11.72	1.83
Traditional two-step filter (1)	21.88	3.53
EKF (1)	23.97	3.56
ATSF (2)	16.03	2.79
Traditional two-step filter (2)	31.06	5.46
EKF (2)	51.05	11.66
ATSF (3)	33.23	10.36
Traditional two-step filter (3)	116.48	46.47
EKF (3)	277.81	125.56

**Table 4:** Computational time and tracking accuracies under different thresholds.

Threshold $\delta_{i}(i\;=\;1,\;2,\;3)$	Computational Time	Tracking Accuracy
$\delta_{1}=0.1$	18.84 s	$RMSE_{l}$ *(m) = 33.23*
		$RMSE_{v}$ *(m/s) = 10.36*
$\delta_{2}=0.01$	212.5 s	$RMSE_{l}$ *(m) = 32.25*
		$RMSE_{v}$ *(m/s) = 9.6*
$\delta_{3}=0.5$	3.71 s	$RMSE_{l}$ *(m) = 68.17*
		$RMSE_{v}$ *(m/s) = 20.1*

## Data Availability

Data sharing not applicable.
